# Supplementary material for: Global hypo-methylation in a proportion of glioblastoma enriched for an astrocytic signature is associated with increased invasion and altered immune landscape
Source: eLife. 2022 Nov 22;11:e77335. doi: 10.7554/eLife.77335 (PMC9681209; doi:10.7554/eLife.77335)
Supplement: Figure 2—source data 1. [file elife-77335-fig2-data1.zip › Figure_2_source_data_1/Figure_2B/homerResults/motif39.similar.html]

motif39

## Information for motif39

T
A
C
G
G
T
A
C
A
C
T
G
C
G
A
T
T
G
A
C
C
T
G
A
C
T
A
G
A
T
C
G
C
T
G
A
T
A
G
C
T
A
G
C
C
A
G
T
A
C
T
G
T
A
C
G
G
T
A
C
C
G
T
A
T
C
A
G
C
T
A
G
T
C
A
G
T
C
G
A
T
C
G
A
C
A
G
T
T
C
A
G
T
A
G
C
C
G
T
A
C
T
A
G
C
G
A
T
C
A
T
G
C
T
G
A
T
A
G
C
G
T
A
C
G
C
A
T
G
T
A
C
C
T
G
A
C
A
T
G
  
Reverse Opposite:  

G
A
T
C
G
A
C
T
C
A
T
G
C
G
T
A
C
A
T
G
A
T
C
G
A
G
C
T
G
T
A
C
G
C
T
A
G
A
T
C
C
G
A
T
A
C
T
G
A
G
T
C
G
T
C
A
A
G
C
T
A
G
C
T
A
G
T
C
A
G
T
C
A
G
T
C
G
C
A
T
A
C
T
G
A
T
G
C
T
G
A
C
G
T
C
A
A
T
C
G
A
T
C
G
G
A
C
T
T
A
G
C
G
A
T
C
G
A
C
T
A
C
T
G
G
C
T
A
T
G
A
C
C
A
T
G
A
T
G
C
  

|  |  |
| --- | --- |
| p-value: | 1e-39 |
| log p-value: | -9.119e+01 |
| Information Content per bp: | 1.798 |
| Number of Target Sequences with motif | 85.0 |
| Percentage of Target Sequences with motif | 3.68% |
| Number of Background Sequences with motif | 2.4 |
| Percentage of Background Sequences with motif | 0.69% |
| Average Position of motif in Targets | 108.7 +/- 52.9bp |
| Average Position of motif in Background | 38.3 +/- 22.5bp |
| Strand Bias (log2 ratio + to - strand density) | -0.2 |
| Multiplicity (# of sites on avg that occur together) | 1.11 |
| Motif File: | file (matrix) reverse opposite |

### Similar de novo motifs found

|  |  |  |  |  |  |  |  |
| --- | --- | --- | --- | --- | --- | --- | --- |
| Rank | Match Score | Redundant Motif | P-value | log P-value | % of Targets | % of Background | Motif file |
| 1 | 0.805 | A C G T T G A C C G T A T A C G C A G T T A C G C T G A C T A G T C A G T A G C T A C G A C G T T A G C C G T A C T A G T A C G T C G A T A G C T A G C C G A T C A T G T A C G T A G C C G T A C T A G C T A G T C A G T C G A T C G A C A G T A T C G T A G C C T G A T C A G C A G T A T C G T C G A T A G C G T A C G A C T | 1e-13 | -30.624987 | 4.07% | 1.71% | motif file (matrix) |
